# Dual strategies for epilepsy management employing pharmacological and non-invasive brain stimulation approaches

**DOI:** 10.3389/fneur.2025.1541064

**Published:** 2025-08-15

**Authors:** Masoud Afshari, Gila Pirzad Jahromi, Mehrdad Roghani

**Affiliations:** ^1^Department of Cognitive Psychology, Institute for Cognitive and Brain Sciences, Shahid Beheshti University, Tehran, Iran; ^2^Neuroscience Research Center, Baqiyatallah University of Medical Sciences, Tehran, Iran; ^3^Neurophysiology Research Center, Shahed University, Tehran, Iran

**Keywords:** epilepsy, non-invasive brain stimulation, transcranial magnetic stimulation, transcranial direct current stimulation, transcranial ultrasound stimulation

## Abstract

Epilepsy is a prevalent neurological disorder that affects more than 50 million individuals worldwide, characterized by seizures, and is often associated with complications such as cognitive impairments, and an increased risk of sudden unexpected death in epilepsy (SUDEP). Despite advancements in pharmacological treatments, one-third of patients develop drug resistance and some experience serious side effects related to drug therapy. This highlights the urgent need for alternative therapeutic approaches. Non-invasive brain stimulation (NIBS) techniques, such as transcranial magnetic stimulation (TMS), transcranial direct current stimulation (tDCS), and transcranial ultrasound stimulation (TUS), have emerged as promising alternatives. These methods modulate brain activity with fewer side effects and show potential for treating drug-resistant epilepsy. However, their clinical application is still limited by factors such as variability in stimulation protocols and patient responsiveness. This review explores the efficacy, underlying mechanisms, and side effects of pharmacological treatments, with a focus on commonly prescribed drugs for epilepsy, as well as selected NIBS techniques, emphasizing their roles in managing epilepsy. By comparing these approaches, we aim to provide insights into optimizing epilepsy treatment strategies and improving patient outcomes. This review suggests that NIBS alone or in combination with pharmacological therapy is a promising method for patients with epilepsy and future research should focus on the effective protocols and related mechanisms.

## 1 Introduction

Epilepsy is a neurological disorders affecting more than 50 million individuals worldwide (1). Its estimated cost in 2019 was around $119 billion in terms of the global economic and healthcare burden (2). It is characterized by seizures and associated neurological dysfunctions that may lead to cognitive deficits, psychological and social challenges, and physical disorders, all of which can impact patients’ quality of life. An estimation showed that around 50% of people with epilepsy experience these comorbidities (3). Furthermore, in some epilepsy cases, a life-threatening condition known as sudden unexpected death in epilepsy (SUDEP) can occur (4).

A range of medications is used to treat epilepsy. These drugs are designed to reduce the onset of seizures and may directly or indirectly impact associated comorbidities resulting from epilepsy. These medications include sodium channel blockers, GABAergic drugs, calcium channel modulators, AMPA receptor antagonists, neurotransmitter modulators, cannabinoids, other medications related to specific disease and valproate sodium. Despite their effectiveness, these drugs often come with various side effects that can affect patients’ quality of life (5). Previous research also mentioned lots of various side effects including drowsiness, dizziness, excessive fatigue, and gastrointestinal disturbances as well as serious conditions like Stevens-Johnson syndrome (6). Many patients may also experience cognitive impairments, concentration difficulties, and mood changes, which can eventually lead to depression, irritability, and anxiety. Drug interactions, dermatological side effects, as well as tolerance and dependence, are other concerns (6). Sodium valproate is another common medication for epilepsy treatment and it has teratogenic effects when taken during pregnancy (7). Additionally, approximately one-third of epilepsy patients suffer from drug-resistant epilepsy (DRE), meaning they do not respond effectively to antiepileptic drugs (8). To overcome this, second- and third-generation anti-epileptic drugs have been introduced in recent decades, which are more tolerable and less toxic and expected to have better efficacy in controlling seizures, especially in patients with DRE, but the evidence is not strong enough yet (9). A recent review discusses new advancements in anti-seizure medications, such as cenobamate and fenfluramine, which may help DRE cases. While these treatments show some effectiveness in reducing seizures and mortality risk, more research is needed to understand their long-term effects on DRE patients (10). Therefore, alternative treatments that are both effective and have fewer side effects for all patients are needed.

Non-invasive brain stimulation (NIBS) has become popular among researchers and clinicians due to its therapeutic potential with fewer side effects. Several common types of NIBS are discussed here, including transcranial magnetic stimulation (TMS), transcranial direct current stimulation (tDCS), and transcranial ultrasound stimulation (TUS). These methods can produce immediate effects on brain function in targeted areas with a single use and long-term effects when applied over several sessions. NIBS can be used as an alternative or with pharmacological therapy and might provide a more effective treatment for epilepsy patients specifically in DRE cases, with fewer side effects.

The objective of this review is to explore both approaches, including commonly used pharmacological treatments and NIBS, and to highlight their efficacy, underlying mechanisms, side effects, and roles in the management of epilepsy.

## 2 Pharmacological treatments in epilepsy

### 2.1 Sodium channel blockers

Phenytoin, lamotrigine, lacosamide, eslicarbazepine acetate (prodrug for S-licarbazepine), fosphenytoin (prodrug for phenytoin), oxcarbazepine (prodrug for licarbazepine), rufinamide, topiramate, zonisamide, cenobamate and carbamazepine (11) are sodium channel blockers. These medications block voltage-gated sodium channels and act as antiepileptic agents (12). Serious adverse effects may occur when patients receive these medications, including ataxia, fatigue, diplopia, drowsiness, dizziness, nausea, and vomiting. Other important and rare complications, include cardiac arrhythmias, hepatotoxicity, lupus-like syndrome, blood dyscrasias, and other complications are possible with these medications (13).

### 2.2 GABA modulators drugs

GABA (gamma-aminobutyric acid) is one of the major inhibitory neurotransmitters and mediates its effects via two GABA_A_ and GABA_B_ receptors (14). GABAergic drugs are used to enhance inhibitory signaling in the brain, reducing neuronal hyperexcitability that leads to seizures (15). Examples of such drugs include first-generation ones like phenobarbital and primidone; second-generation like benzodiazepines; and newer-generation like topiramate, felbamate, retigabine (which also affects voltage-gated K^+^ channels (11)), Cenobamate and stiripentol, which are prescribed based on the type of epilepsy and patient characteristics (11, 16). Also, vigabatrin is a GABA-transaminase inhibitor, which results in reduced GABA metabolism and increased its concentration in the brain. Tiagabine is another medication that acts as a reuptake inhibitor of GABA in neurons and glia (11). However, in some medications, side effects such as sedation, cognitive impairment, tolerance, and dependency limit their long-term use (11, 16). Sometimes, during excessive activation of the GABA_A_ receptor, outflow of bicarbonate leads to neuronal depolarization and may cause seizures. Carbonic anhydrase inhibitors such as acetazolamide may reduce seizure activity in some epilepsy cases, but tolerance is the main side effect. Topiramate, zonisamide, and possibly lacosamide are other proposed alternatives that also utilize this mechanism (11). GABA disposition may also be utilized by some drugs, such as gabapentin and topiramate (11).

### 2.3 Sodium valproate

Sodium valproate is a widely used anti-epileptic drug. It works by stabilizing electrical activity in the brain, preventing seizures. One of the mechanisms involves increasing GABA activity. By increasing GABA activity, this medication reduces abnormal electrical activity that leads to seizures. It also inhibits sodium channels and T-type calcium channels. However, serious but rare side effects include liver toxicity, pancreatitis, and teratogenic effects (if taken during pregnancy) (17).

### 2.4 Calcium channel modulators

Drugs like Ethosuximide and Methsuximide are calcium channel modulators (T-type) (16). These medications modulate the entry of calcium ions (Ca^2+^) into neurons through voltage-gated calcium channels (18). By reducing neuronal excitability, these medications prevent abnormal electrical activity that can lead to seizure attacks.

### 2.5 AMPA (α-amino-3-hydroxy-5-methyl-4-isoxazolepropionic acid) receptor antagonists

AMPA receptors are a subtype of ionotropic glutamate receptors responsible for fast excitatory synaptic transmission in the brain (19). In epilepsy, excessive glutamate signaling through these receptors can increase neuronal excitability and cause seizures (20, 21). A common drug in this family is perampanel: a non-competitive AMPA receptor antagonist used to treat partial-onset and generalized tonic–clonic seizures (20). Riluzole, memantine, and ketamine are examples of NMDA receptor antagonists that may also be useful in controlling seizures related to glutamate signaling (20).

### 2.6 Neurotransmitter modulators

Changes in neurotransmitter release at synapses may affect the brain activity and also may influence seizures attacks. For instance, Lamotrigine is a selective glutamate release inhibitor due to its effect on sodium and calcium channels. Levetiracetam and brivaracetam have more direct effects on neurotransmitters release and their primary targets for binding are synaptic vesicle protein 2A (SV2A). This protein is found in presynaptic neurons and plays a role in synaptic release (11).

### 2.7 Cannabinoids

Cannabidiol (CBD) is another drug that has gained attention due to its anti-epileptic activity in certain types of epilepsy such as Dravet and Lennox–Gastaut syndrome. Although the mechanism of action of CBD in reducing seizures is not well understood (11), one of the possible mechanisms is that CBD is an antagonist of GPR55 receptors, resulting in reduced intracellular Ca^2+^ leading to reduced neural excitability. It also blocks T-type Calcium channels (22). Generally, CBD is a well-tolerated drug but some common side effects such as decreased appetite, diarrhea and increased liver enzymes may occur and also some serious but rare side effects such as pneumonia, liver failure and status epilepticus may happen depending on the patient’s condition (22).

### 2.8 Other medications related to specific diseases

Sometimes epilepsy results from another abnormality. Cortical development malformation is one of the common causes of epileptic encephalopathies which may be related to mTOR (mechanistic target of rapamycin) pathway. mTOR inhibitors such as everolimus and sirolimus have been effective in some investigations. Cerliponase alfa also is another drug that may be effective in seizures resulting from Batten disease (neuronal ceroid lipofuscinosis) (11).

### 2.9 Drug resistance in epilepsy (DRE)

In DRE individuals, morbidity and mortality rates increase, and they are more likely to develop psychiatric problems, and therefore quality of life decreases (23). The proportion of DRE patients has not changed over the past decades. One of the alternatives is surgery, which is invasive and may cause permanent complications that reduce quality of life. Furthermore, pharmacological therapies are not regional and affect other brain areas as well, causing many side effects (24). These limitations underscore the need for alternative therapeutic strategies and methods.

## 3 Non-invasive brain stimulation (NIBS) techniques

### 3.1 Transcranial magnetic stimulation (TMS)

TMS is a form of NIBS (Table 1) that stimulates the brain cortex with magnetic pulses with different intensities and frequencies (25). The mechanism of TMS involves inducing an electrical field in the targeted brain area (26). Low-frequency repetitive TMS (LF-rTMS) is a common protocol of TMS that has inhibitory effects on the brain, while high-frequency rTMS (HF-rTMS) has the opposite effect (27). The idea behind TMS in epilepsy treatment is that it can reduce cortical hyperexcitability and result in decreased seizure frequency in epilepsy patients. A meta-analysis has shown that LF-rTMS may be effective in DRE cases (28).

**Table 1:** This table compares three different non-invasive brain stimulation (NIBS) approaches for epilepsy management based on their basic mechanisms, applications, and cellular mechanisms.

	Transcranial magnetic stimulation (TMS)	Transcranial direct current stimulation (tDCS)	Transcranial ultrasound stimulation (TUS)
Mechanism	Induces an electrical field Affects neurons in the brain High-frequency rTMS: excitatory Low-frequency rTMS: inhibitory	Modulates neuronal membrane potentials Anodal tDCS: excitatory Cathodal tDCS: inhibitory	Focuses ultrasound waves on specific brain regions Modulates neural activity
Applications	May reduce cortical hyperexcitability and decrease seizure frequency	Reduces seizure frequency in drug-resistant focal epilepsy	Reduces seizure frequency Improves anxiety, depression, and social behaviors Enables targeted drug delivery via BBB opening
Cellular mechanism	GABA-A receptor modulation Improved immune function Reduce neural excitability Ion channel modulation Improved Synaptic plasticity etc…	Modulate neuroinflammation Modulate neurotrophin levels Modifies EEG patterns Decreases hyperexcitability Reduces mossy fiber sprouting Reduces BDNF overexpression	Inhibits neuronal apoptosis (↑ Bcl-2, ↓ Bax, caspase-3) Modulates neuroinflammation Opens BBB

A recent study suggests that targeting both sides of the cerebellum with continuous theta burst stimulation (cTBS), consisting of three stimulus pulses (50 Hz repeated at 5 Hz, totaling 600 stimuli in 40 s), can be beneficial in DRE individuals. The cerebellum was stimulated twice at 5-min intervals, daily for two working weeks (29). Another study also showed that a 2-week treatment with LF-rTMS reduces the number of seizures in patients with benign epilepsy (30). Additionally, another study used LF-rTMS for 10 days, targeting the central region of the brain (C5 or C6) in self-limited epilepsy, and found that it can improve the excitation-inhibition (E-I) imbalance with favorable outcomes (31).

Several studies have explored the molecular mechanisms of rTMS in epilepsy. In a mouse model of status epilepticus, low-frequency rTMS at 0.5 Hz (600 pulses, 20% intensity, for 20 min twice daily over 5 days) was found to have beneficial effects by regulating GABA_A_ receptor activity (a target of GABAergic drugs), improving immune function, and modulating biological processes (32). Additionally, low-frequency rTMS (300 pulses daily at 40% intensity for 28 days at frequencies of 0.3 Hz, 0.5 Hz, or 1 Hz depending on the experimental group) significantly reduced spontaneous recurrent seizures in rats with medial temporal lobe epilepsy, increasing AMPA receptor expression and restoring synaptic plasticity in the hippocampus and also improving cognitive function (33). In a picrotoxin-induced epilepsy model in mice, low-frequency rTMS (10 sessions, varying frequencies (0.5–1 Hz) and intensity) significantly reduced seizure number and severity, likely by modulating the E-I balance of neurons (34).

In a review, other cellular mechanisms of TMS in epilepsy have been proposed, including changes in neural excitability, ion channel modulation, alterations in synaptic function, and ephaptic effects (alterations in communication between neurons through electric fields, rather than synaptic transmission) (35).

It was also reported in a recent review that NIBS such as TMS and tDCS are generally safe and promising in pediatric epilepsy but also more research is needed to find a suitable protocol and validate its long-term efficacy (36).

### 3.2 Transcranial direct current stimulation (tDCS)

The tDCS is another potential therapeutic option for epilepsy management. Like TMS, tDCS is a form of NIBS but operates via a different mechanism. Typically, two electrodes (cathode and anode) are placed on the scalp, with cathodal stimulation (c-tDCS) showing inhibitory effects and anodal stimulation (a-tDCS) having excitatory effects (37). A meta-analysis has shown that c-tDCS appears particularly promising for drug-resistant focal epilepsy (38). Another recent review of RCTs also showed that tDCS is safe for DRE individuals and can reduce seizure frequency (39). Another meta-analysis reported effectiveness in reducing seizure frequency but not in decreasing epileptiform discharges (40).

A study on drug-resistant focal epilepsy patients using tDCS (2 mA cathodal stimulation on the seizure target zone for 30 min over 2 weeks [10 days]) reported a positive effect on seizure frequency (41). Another study on medication-refractory focal epilepsy patients showed that c-tDCS treatment for 2 weeks, on brain areas based on the patient’s seizure focus, reduced seizure frequency with worsening in one case (42).

In a pentylenetetrazole (PTZ)-induced kindling model of epilepsy in rats, c-tDCS, either alone or in combination with diazepam, modulated neurotrophin and neuroinflammatory responses. Specifically, it decreased interleukin-1 beta (IL-1β) levels in the hippocampus while increasing IL-1β levels in the cortex, without significant effect on seizure activity. tDCS was applied daily for 20 min over 10 days using a 0.5 mA current (current density: 33.4 A/m^2^), with the cathodal electrode placed over the parietal cortex and the anodal electrode over the supraorbital area (43). In a kainic acid-induced status epilepticus rat model, c-tDCS (1 mA/3.14 mm^2^, 30 min/day for 5 days over the dorsal hippocampus) was applied and the severity of seizures significantly reduced, altering EEG patterns, suggesting reduced brain hyperexcitability. Follow-up showed tDCS reduced adverse outcomes such as mossy fiber sprouting and BDNF overexpression, highlighting its therapeutic potential for epilepsy (44).

### 3.3 Transcranial ultrasound stimulation (TUS)

TUS involves focusing ultrasound waves on specific brain areas to alter neural activity. In one study, ultrasound neuromodulation (1 kHz PRF, 50% duty cycle, 1 s burst duration, 4 s inter-stimulus interval, 30 min/day for 7 days targeting the left cortex and hippocampus) significantly prolonged seizure latency and improved anxiety-like behaviors in kainic acid (KA)-induced epileptic mice. The treatment also inhibited neuronal apoptosis by upregulating anti-apoptotic protein Bcl-2 and downregulating pro-apoptotic proteins Bax and caspase-3, as well as reducing inflammation markers such as IL-1β, TNF-α, and astrocyte and microglial markers (45). Another study found that ultrasound stimulation reduced seizure activity and improved social and depressive related behaviors in a mouse model of mesial temporal lobe epilepsy induced by kainic acid (46).

In a different approach, ultrasound was used in combination with drugs to induce non-invasive brain lesions for epilepsy treatment. In a pilocarpine-induced epilepsy model, researchers used magnetic resonance-guided low-intensity focused ultrasound to open the blood–brain barrier in the hippocampus, allowing a neurotoxin (quinolinic acid) to enter and cause targeted neuronal damage. The method significantly reduced seizure activity (47). Another study developed closed-loop wearable ultrasound deep brain stimulation (UDBS) system to suppress seizures by targeting the hippocampus. This system showed promise in detecting and controlling seizures in a mouse model of epilepsy (48). Lastly, both low-intensity pulsed ultrasound (LIPUS) and low-intensity continuous ultrasound (LICUS) have been shown to effectively suppress seizure attacks in a kainite-induced temporal lobe epilepsy (TLE) model by reducing neural oscillations in the hippocampus (49).

A pilot study on transcranial focused ultrasound stimulation for temporal lobe epilepsy suggests it is largely safe, with no significant histopathologic damage observed in participants. However, a notable decline in verbal memory post-treatment raises concerns about potential cognitive effects (50). Another study provides initial evidence on the safety and feasibility of anterior nucleus of the thalamus (ANT) focused ultrasound ablation (FUSA). While seizure reduction was observed, in one patient verbal fluency and memory impairments emerged as a potential concerns (51). In another pilot study, six patients with mesial temporal lobe epilepsy (mTLE) received six TUS sessions (two per week) targeting the hippocampus. Patients experienced seizure reduction, with effects lasting from weeks to several months. No adverse effects were reported (52).

## 4 Other methods

### 4.1 Acupuncture

Acupuncture is a minimally invasive and relatively safe technique in traditional Chinese medicine. Traditionally, in this method, needles are inserted in certain points of the body that can lead to neuromodulation with stimulation of peripheral-central circuit (53). Previous studies have shown the anti-epileptic mechanism behind this method is mainly related to anti-inflammatory effect, anti-apoptosis effect and neuroendocrine and neurotransmitter regulation (54).

The combination of acupuncture with pharmacological treatments may have some beneficial effects in patients suffering from epilepsy (55). It also reported in a review that both manual and electroacupuncture showed this method effective in epilepsy in research and mentioned the effectiveness are also similar to other neuromodulation techniques used in DRE (53). It was also reported patients with temporal lobe epilepsy who underwent acupuncture treatment for 10 weeks, reduced the number of seizures and improved quality of life (56).

## 5 Discussion and conclusion

The role of pharmacological treatments in managing epilepsy is important and could significantly improve patients’ quality of life. However, DRE remains a major challenge, with many patients not responding to pharmacological therapies. NIBS techniques offer a promising and safe alternative, either as independent treatments or in combination with anti-epileptic drugs (Table 2). Traditional medicine like acupuncture also sounds promising as another neuromodulation method (53). However, despite all the advantages, some limitations also exist. For example, TMS mostly affects the cortex, and it is difficult to reach deeper brain regions. Even in the targeted area, it cannot discriminate which type of neuron (excitatory or inhibitory) is being stimulated (57). Also, the high cost of TMS, lack of standardization and variability in patient response to this treatment remain limitations (36). More research is needed to fully understand the mechanisms, establish long-term efficacy, and develop personalized and standardized protocols or explore new techniques such as Transcranial Burst Electrical Stimulation (tBES) (58) for effective use of NIBS in patients with epilepsy.

**Table 2:** Comparison of drug treatment and non-invasive brain stimulation (NIBS) across different aspects of epilepsy management.

	Pharmacological treatments	Non-invasive brain stimulation (NIBS)
Mechanism of the treatment	Alters neurotransmitter activity, ion channels, and receptors to reduce seizure activity	Modulates cortical excitability and brain networks to suppress hyperactivity
Examples	Sodium channel blockers, GABAergic drugs, AMPA receptor antagonists, calcium channel modulators, neurotransmitters modulators, cannabinoids, other medications related to specific disease	TMS, tDCS, TUS
Effectiveness	Effective for most patients but limited in drug-resistant epilepsy	Promising results, particularly for drug-resistant cases
Side effects	Drowsiness, dizziness, cognitive impairment, metabolic issues, teratogenicity, dependence	Mild headaches, scalp discomfort, rare cases of seizure induction
Long-term impact	Chronic use required; some patients develop tolerance and dependence	Potential for long-term neuroplastic changes with sustained effects
Suitability for DRE	Limited; one-third of patients remain resistant	More effective in drug-resistant epilepsy
Impact on comorbidities	Can address psychological issues but may also exacerbate them	May improve cognition and mood disorders
Invasiveness	Systemic effects throughout the body	Non-invasive, localized to targeted brain regions
